# Long-Term Health Effects of Neutering Dogs: Comparison of Labrador Retrievers with Golden Retrievers

**DOI:** 10.1371/journal.pone.0102241

**Published:** 2014-07-14

**Authors:** Benjamin L. Hart, Lynette A. Hart, Abigail P. Thigpen, Neil H. Willits

**Affiliations:** 1 Department of Anatomy, Physiology and Cell Biology, School of Veterinary Medicine, University of California Davis, Davis, California, United States of America; 2 Department of Population Health and Reproduction, School of Veterinary Medicine, University of California Davis, Davis, California, United States of America; 3 Department of Statistics, University of California Davis, Davis, California, United States of America; Utah State University, United States of America

## Abstract

Our recent study on the effects of neutering (including spaying) in Golden Retrievers in markedly increasing the incidence of two joint disorders and three cancers prompted this study and a comparison of Golden and Labrador Retrievers. Veterinary hospital records were examined over a 13-year period for the effects of neutering during specified age ranges: before 6 mo., and during 6–11 mo., year 1 or years 2 through 8. The joint disorders examined were hip dysplasia, cranial cruciate ligament tear and elbow dysplasia. The cancers examined were lymphosarcoma, hemangiosarcoma, mast cell tumor, and mammary cancer. The results for the Golden Retriever were similar to the previous study, but there were notable differences between breeds. In Labrador Retrievers, where about 5 percent of gonadally intact males and females had one or more joint disorders, neutering at <6 mo. doubled the incidence of one or more joint disorders in both sexes. In male and female Golden Retrievers, with the same 5 percent rate of joint disorders in intact dogs, neutering at <6 mo. increased the incidence of a joint disorder to 4–5 times that of intact dogs. The incidence of one or more cancers in female Labrador Retrievers increased slightly above the 3 percent level of intact females with neutering. In contrast, in female Golden Retrievers, with the same 3 percent rate of one or more cancers in intact females, neutering at all periods through 8 years of age increased the rate of at least one of the cancers by 3–4 times. In male Golden and Labrador Retrievers neutering had relatively minor effects in increasing the occurrence of cancers. Comparisons of cancers in the two breeds suggest that the occurrence of cancers in female Golden Retrievers is a reflection of particular vulnerability to gonadal hormone removal.

## Introduction

In the last three decades, the practice of spaying female dogs and castrating males (both referred to herein as neutering) has greatly increased. The current estimate is that in the U.S., 83 percent of all dogs are neutered [1] and, increasingly, neutering is being performed prior to 6 mo., as advocated by many veterinarians and animal activists. The impetus for this widespread practice is presumably pet population control, and the belief that mammary gland and prostate cancers are prevented and aggressive male behavior is markedly less likely than in those neutered later. This societal practice in the U.S. continues to contrast with the general attitudes in many European countries, where neutering is commonly avoided and not promoted by animal health authorities [2]–[4].

In the last decade or so, studies have pointed to some of the adverse effects of neutering in dogs on several long-term health parameters by looking at one disease syndrome in one breed or in pooling data from several breeds. With regard to cancers, a study on osteosarcoma (OSA) in several breeds found a 2-fold increase in neutered dogs relative to intact dogs [5], and in Rottweilers neutering prior to 1 year of age was associated with an increased occurrence of OSA to 3–4 times that of intact dogs [6].

A study of cardiac hemangiosarcoma (HSA) in spayed females found that the incidence of this cancer was 4 times greater than that of intact females [7] and another on splenic HSA in spayed females found rates 2 times greater than of intact females [8]. A study on lymphosarcoma (lymphoma, LSA) found that neutered females had a higher incidence of the disease than intact females [9]. Cutaneous mast cell tumors (MCT) were studied in several dog breeds revealing an increase in incidence in neutered females to 4 times that of intact females [10]. Another cancer of concern is prostate cancer that, in contrast to humans, is potentiated by the removal of testosterone. One extensive study found that this cancer occurred in neutered males 4 times as frequently as in intact males [11].

The most frequently mentioned advantage of early neutering of female dogs is protection against mammary cancer (MC) [12]. However, a recent meta-analysis of published studies on neutering females and MC found that the evidence linking neutering to a reduced risk of MC is weak [13].

Three very recent studies are particularly relevant in the discussion of neutering and cancers. One was a comprehensive study, from this center, on neutering in 759 Golden Retrievers where males were compared with females and effects of neutering were evaluated in early-neutered (<1 year), late-neutered (>1 year) and intact dogs [14]. Almost 10 percent of early-neutered males were diagnosed with LSA, 3 times more than intact males. There were no cases of MCT in intact females, but in late-neutered females the rate was nearly 6 percent. The incidence of HSA in late-neutered females was also higher than that of intact females. The occurrence of MC was very low and was only seen in a couple of late-neutered females.

A study utilizing the Veterinary Medical Database of over 40,000 dogs found that neutered males and females were more likely to die of cancer than intact dogs, especially of OSA, LSA and MCT [15]. This study included no information on age of neutering. The most recent publication in this area is a study of Vizslas utilizing owner-reported disease occurrence in an online survey, in which the incidence of cancers was reported higher in neutered dogs than in intact dogs [16]. The main cancers related to neutering were LSA, HSA and MCT. The occurrence of MC was very low in females left intact.

With regard to joint disorders, one study of effects of neutering in larger breeds documents a 3-fold increase in excessive tibial plateau angle – a known risk factor for development of cranial cruciate ligament tears or rupture (CCL) [17]. Across several breeds, a study of CCL found that neutered males and females were 2 to 3 times more likely than intact dogs to have this disorder [18]. Neither study examined early versus late neutering with regard to this disorder. The study from this center of neutering in Golden Retrievers (mentioned above with regard to cancers [14]) included examination of joint disorders. Of the early-neutered males, 10 percent were diagnosed with hip dysplasia (HD), double the occurrence of that in intact males. There were no cases of CCL diagnosed in intact males or females, but in early-neutered males and females the occurrences were 5 percent and 8 percent, respectively.

One factor that merits attention with regard to the effects of neutering on joint disorders relates to documented effects of neutering in increasing body weight [19], as reflected in body condition score (BCS). Additional weight on the joints is considered to play a role in the onset of joint disorders [19], [20]. While neutering is expected to increase BCS, the issue of concern here is whether neutered dogs with a joint disorder have consistently higher BCSs at the time of diagnosis than do neutered dogs without the joint disorder in the same age range. In the previous analyses on Goldens [14] there was no consistent and major difference in BCS between early neutered dogs with and without a joint disorder. For dogs diagnosed with a joint disorder, some increase in BCS would be expected as a function of less activity due to discomfort from painful joints. Therefore, a modestly higher BCS was predicted for neutered dogs with a joint disorder than in the neutered counterparts without a joint disorder.

The above study on Golden Retrievers [14] raised a major question about breed differences in the effects of neutering, which are relevant for breeders and caregivers of puppies when deciding if, and when, to neuter. A more basic issue concerns insights into the possible pathogenic factors triggering the occurrence of the cancers under consideration. The present study, using the same veterinary hospital database, explored the effects of neutering on joint disorders and cancers in the popular Labrador Retriever to compare with the Golden Retriever, with an addition of several years to the database. The age periods of neutering were refined as <6 mo., 6–11 mo., 12–23 mo. (1 year), and 2 through 8 years to provide more detailed information on the effects of gonadal hormone removal. The Golden is known for being particularly vulnerable to cancers [21], so we expected some major differences from the Labrador where cancer-related deaths are less frequent than in Goldens [21].

In addition to reporting on the incidence of the individual joint disorders and cancers, a new slant on analyses in the present study combined the incidence of all three joint disorders that have shown evidence of being increased by neutering (HD, CCL, and elbow dysplasia, ED) for one data-point representing the incidence of dogs diagnosed with at least one of the joint disorders, after controlling for multiple diagnoses. This analysis was based on the perspective that for dog owners or breeders, avoidance of any of the debilitating joint disorders would be of prime interest. This analysis was also deemed logical for pathophysiological reasons because a disruption of the growth plate closure by gonadal hormone removal in the joint developmental stage would be expected to apply to all the joint disorders. The study also combined the incidence of dogs diagnosed with at least one of the cancers (LSA, HSA, MCT) for one data point, after controlling for multiple diagnoses, because for dog owners avoidance of any of the cancers would be important. This analysis seemed logical, as there may be a common factor involved in increasing these three particular cancers in neutered dogs because these cancers are repeatedly reported as being increased by neutering in several studies.

## Methods

### Ethics Statement

No animal care and use committee approval was required because, in conformity with campus policy, the only data used were from retrospective veterinary hospital records. Upon approval, faculty from the University of California, Davis (UCD), School of Veterinary Medicine, are allowed use of the record system for research purposes by the Veterinary Medical Teaching Hospital (VMTH). The co-authors of this study were given permission by the VMTH to use their veterinary hospital records for this study.

### Data Collection

The dataset used in this study was obtained from the computerized hospital record system (Veterinary Medical and Administrative Computer System) of the Veterinary Medical Teaching Hospital (VMTH) at UCD. The subjects included were gonadally intact and neutered female and male Labrador Retrievers and Golden Retrievers, from 1 through 8 years of age and admitted to the hospital between January 1, 2000 and December 31, 2012, for 13 years of data. If a disease of interest occurred before 12 months of age or before January 1, 2000, that case was removed for that specific disease analysis, but included in other disease analyses.

Data on patients at 9 years of age or older were not considered. This was deemed an appropriate cut-off point in order to exclude disease information on advanced-aged dogs where the effects of aging would confound interpreting the disease effects related to neutering. Additional inclusion criteria were requirements for information on date of birth, age at neutering (if neutered) and age of diagnosis (or onset of clinical signs) of the joint disorder or cancer. The age at neutering was classified as <6 mo., 6–11 mo., 1 year (12 - <24 mo.), and 2–8 years (2 - <9 years). For all neutered dogs, the neuter status at the time of each visit was reviewed to ensure that neutering occurred prior to onset of the first clinical signs or diagnosis of any disease of interest. If a disease of interest occurred before neutering, the diseased dog was recorded as intact for that specific disease analysis. For the same dog where a different disease occurred after neutering, the dog was recorded as neutered for that disease analysis. Detailed reviews of patient records were performed for evidence of disease occurrence meeting specific diagnostic criteria (see below). Using this screening, only diseases with at least 15 cases in the database were included in the study.

For both breeds, many cases with neutering did not include detailed data on age at neutering. With a very large database for the Labrador, there was a sufficient number of dogs with these data to restrict the analyses to cases for which the age at time of neutering was available from the record system. For the Golden with fewer cases, where additional neutering date information was necessary, telephone calls to the referring veterinarians were made to obtain the neutering dates for case patients born after 2000. Because of the number of neutered dogs where age at neutering was not available from either the record or by phone call, there were proportionately more intact cases in the final data set than would be expected in the population at large.

Golden Retriever cases with complete data for analyses totaled 1,015, with 543 males (315 neutered and 228 intact) and 472 females (306 neutered and 166 intact). Labrador Retriever cases with complete data for analyses totaled 1,500 cases with 808 males (272 neutered and 536 intact) and 692 females (347 neutered and 345 intact). The number of cases analyzed for each disease varied somewhat among diseases because a case could be excluded for one disease analysis, if the diagnosis was made prior to 1 year of age, was unconfirmed, or was outside of study range, but would be included for other diseases if no diagnosis was made or where the diagnoses were confirmed after 1 year of age and within the study range.

Table 1 defines the categories of diagnoses based on information in the record of each case. A patient was considered as having a disease of interest if the diagnosis was made at the VMTH or by a referring veterinarian and later confirmed at the VMTH. Patients diagnosed with HD, ED and/or CCL presented with clinical signs such as difficulty moving, standing up, lameness, and/or joint pain; diagnoses were confirmed with radiographic evidence, orthopedic physical examination and/or surgical confirmation. Diagnoses of the various cancers (LSA, HSA, MCT, MC) were accompanied by clinical signs such as enlarged lymph nodes, lumps on the skin or presence of masses, and confirmed by imaging, appropriate blood cell analyses, chemical panels, histopathology and/or cytology. Pyometra was confirmed by ultrasonic evidence and/or post-surgically after removal of the uterus. When a diagnosis was listed in the record as “suspected” based on clinical signs, but the diagnostic tests were inconclusive, the case was excluded from the analysis for that specific disease, but included for other diseases.

**Table 1:** Categories used in determining diagnosis for joint disorders and cancers of interest in Golden Retrievers and Labrador Retrievers (1–8 years old) admitted to the Veterinary Medical Hospital, University of California, Davis, from 2000–2012.

Classification	Definition
No disease	No evidence of a joint disorder or cancer of interest in the medical records
VMTH	Diagnosed at the VMTH
Referring Veterinarian/VMTH	Diagnosed by referring veterinarian and confirmed at the VMTH through treatment or further testing
Referring Veterinarian	Diagnosed by referring veterinarian but no confirming diagnostic tests done at the VMTH. Unconfirmed cases were excluded from analysis for the specific joint disorder or cancer
Invalid (suspected)	Diagnosis was suspected based on clinical signs, but diagnostic tests were inconclusive or not done. Unconfirmed cases were excluded from analysis for the suspected joint disorder or cancer
Invalid (confirmed)	Diagnosed prior to January 2000 or before 1 year of age. Invalid cases were excluded from analysis for the specific joint disorder or cancer.

**Figure 1:**
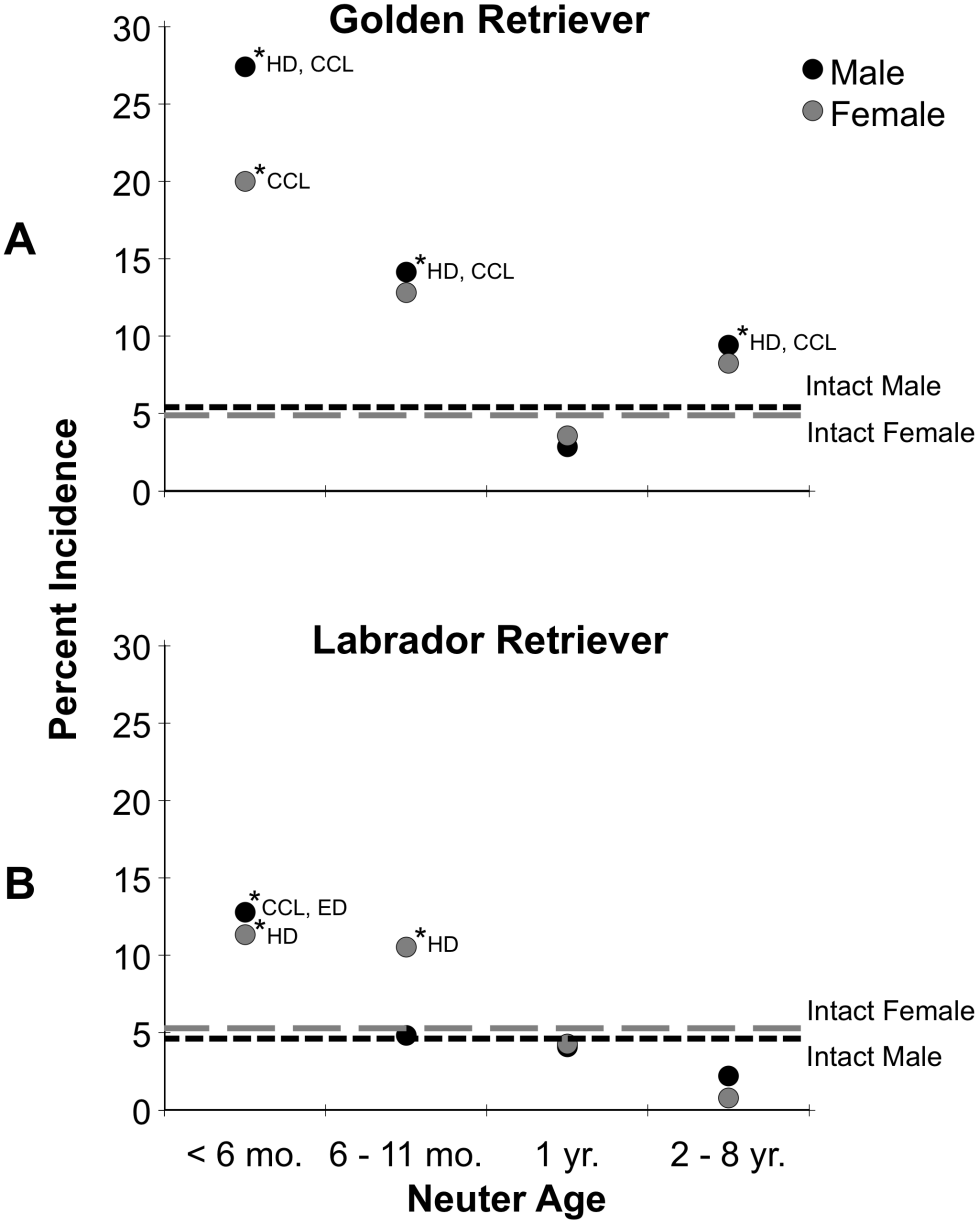
Incidence of the occurrence of at least one joint disorder in male and female Golden Retrievers (top) and Labrador Retrievers (bottom), as a function of age at neutering. The occurrences in intact males and females for the same measure are shown by the horizontal lines. The asterisks indicate significance from the intact level, and the abbreviations reveal the joint disorders contributing to the dots when significant.

**Figure 2:**
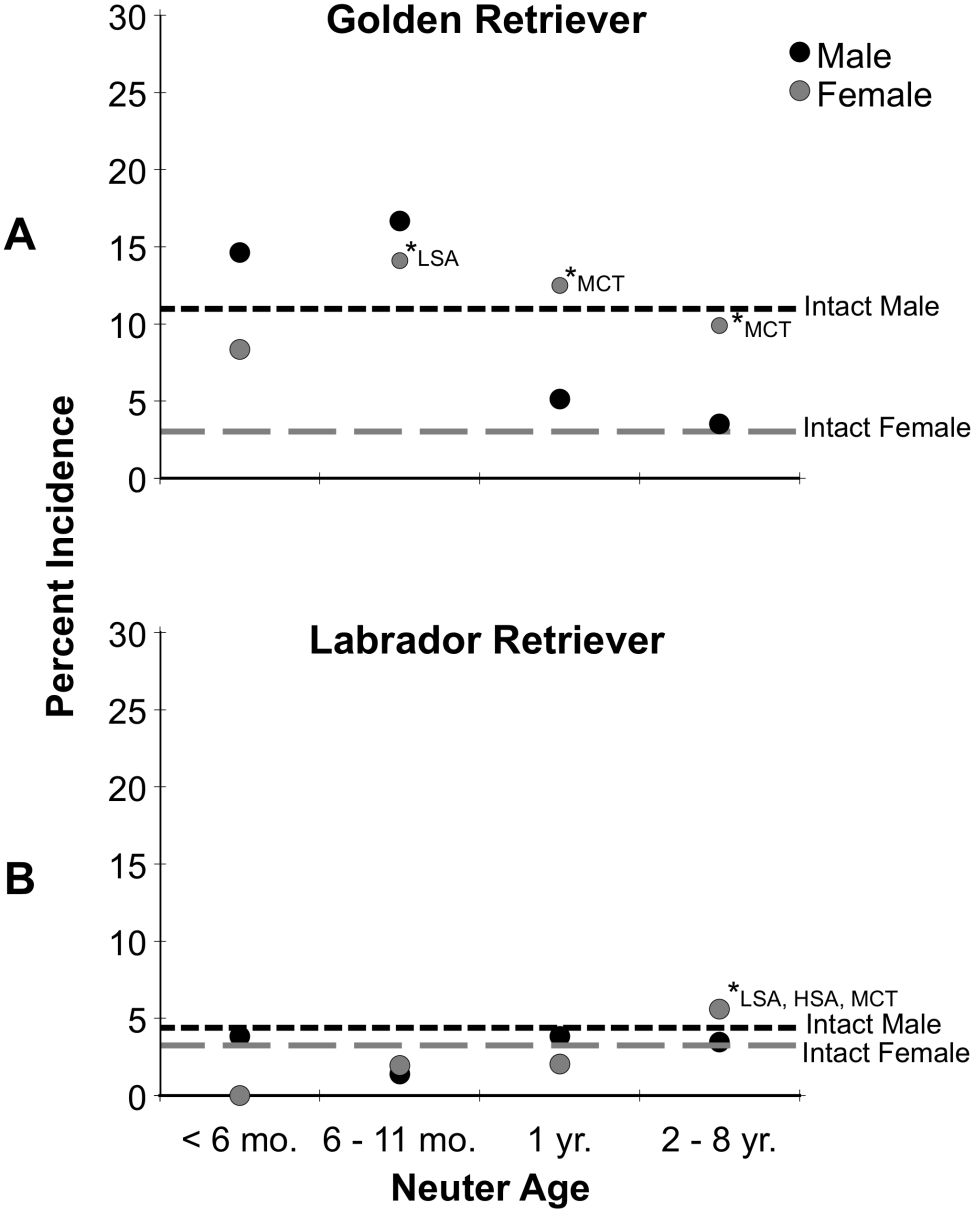
Incidence of the occurrence of at least one cancer in male and female Golden Retrievers (top) and Labrador Retrievers (bottom), as a function of age at neutering. The occurrences in intact males and females for the same measures are shown by the horizontal lines. The asterisks indicate significance from the intact level, and the abbreviations reveal the cancers contributing to the dots when significant.

The analyses used in Figures 1 and 2 portray single data-points representing the incidence of dogs diagnosed with at least one joint disorder or at least one cancer, after controlling for multiple diagnoses. The data for incidence of individual joint disorders and cancers are presented in Tables 2 through 5.

**Table 2:** Golden Retriever males and females, joint disorders.

	HD	CCL	ED
Male <6 months	**11/75 (14.67)**	**8/89 (8.99)**	5/84 (5.95)
Male 6–11 months	**9/113 (7.96)**	**4/123 (3.25)**	4/116 (3.45)
Male 1 year	1/38 (2.63)	0/41 (0)	0/38 (0)
Male 2–8 years	**4/55 (7.27)**	**2/59 (3.39)**	0/59 (0)
Male Intact	9/221 (4.07)	0/226 (0)	5/222 (2.25)
Female <6 months	9/92 (9.78)	**11/101 (10.89)**	0/97 (0)
Female 6–11 months	4/79 (5.06)	**4/81 (4.94)**	3/81 (3.7)
Female 1 year	0/30 (0)	0/32 (0)	1/30 (3.33)
Female 2–8 years	4/86 (4.65)	**3/89 (3.37)**	0/88 (0)
Female Intact	6/163 (3.68)	0/165 (0)	2/164 (1.22)

For ages 1 through 8 years, for each neuter period, the joint disorders are: hip dysplasia (HD), cranial cruciate ligament tear or rupture (CCL), and elbow dysplasia (ED). Shown are number of cases over number in the pool, with percentages given in parentheses. When bolded the incidence is significantly above that of intact dogs.

**Table 3:** Golden Retriever males and females, cancers.

	LSA	MCT	HSA
Male <6 months	6/89 (6.74)	3/90 (3.33)	5/90 (5.56)
Male 6–11 months	**14/122 (11.48)**	4/124 (3.23)	2/122 (1.64)
Male 1 year	0/41 (0)	1/40 (2.5)	1/39 (2.56)
Male 2–8 years	0/58 (0)	2/60 (3.33)	0/59 (0)
Male Intact	9/226 (3.98)	8/225 (3.56)	8/220 (3.64)
Female <6 months	4/98 (4.08)	**3/102 (2.94)**	1/102 (0.98)
Female 6–11 months	**9/82 (10.98)**	1/81 (1.23)	1/79 (1.27)
Female 1 year	2/32 (6.25)	**1/32 (3.13)**	1/32 (3.13)
Female 2–8 years	1/84 (1.19)	**5/88 (5.68)**	2/84 (2.38)
Female Intact	3/166 (1.81)	0/165 (0)	2/165 (1.21)

For ages 1 through 8 years, for each neuter period, the cancers are: lymphosarcoma (LSA), mast cell tumor (MCT), and hemangiosarcoma (HSA). Shown are number of cases over number in the pool, with percentages given in parentheses. When bolded the incidence is significantly above that of intact dogs.

**Table 4:** Labrador Retriever males and females, joint disorders.

	HD	CCL	ED
Male <6 months	0/48 (0)	**4/53 (7.55)**	**2/48 (4.17)**
Male 6–11 months	1/68 (1.47)	2/72 (2.78)	0/67 (0)
Male 1 year	1/50 (2.00)	1/52 (1.92)	0/49 (0)
Male 2–8 years	0/92 (0)	0/93 (0)	**2/93 (2.15)**
Male Intact	9/528 (1.7)	12/531 (2.26)	3/525 (0.57)
Female <6 months	**3/56 (5.36)**	3/59 (5.08)	1/57 (1.75)
Female 6–11 months	**5/99 (5.05)**	5/101 (4.95)	0/103 (0)
Female 1 year	**2/47 (4.26)**	0/50 (0)	0/50 (0)
Female 2–8 years	0/131 (0)	1/128 (0.78)	0/132 (0)
Female Intact	6/345 (1.74)	8/343 (2.33)	4/343 (1.17)

For ages 1 through 8 years, for each neuter period, the joint disorders are: hip dysplasia (HD), cranial cruciate ligament tear or rupture (CCL), and elbow dysplasia (ED). Shown are number of cases over number in the pool, with percentages given in parentheses. When bolded the incidence is significantly above that of intact dogs.

**Table 5:** Labrador Retriever males and females, cancers.

	LSA	MCT	HSA
Male <6 months	0/52 (0)	2/53 (3.77)	0/53 (0)
Male 6–11 months	0/72 (0)	0/73 (0)	1/73 (1.37)
Male 1 year	1/52 (1.92)	0/51 (0)	1/51 (1.96)
Male 2–8 years	0/93 (0)	2/89 (2.25)	1/93 (1.08)
Male Intact	4/530 (0.75)	12/533 (2.25)	7/531 (1.32)
Female <6 months	0/59 (0)	0/60 (0)	0/60 (0)
Female 6–11 months	0/104 (0)	2/103 (1.94)	0/104 (0)
Female 1 year	0/49 (0)	1/50 (2)	0/50 (0)
Female 2–8 years	2/131 (1.53)	5/126 (3.97)	0/133 (0)
Female Intact	4/342 (1.17)	6/344 (1.74)	1/345 (0.29)

For ages 1 through 8 years, for each neuter period, the cancers are: lymphosarcoma (LSA), mast cell tumor (MCT), and hemangiosarcoma (HSA). Shown are number of cases over number in the pool, with percentages given in parentheses. When bolded the incidence is significantly above that of intact dogs.

Given that body weights are difficult to compare among dogs because of the confounding factor of variations in body height, BCSs were used. The BCS system used by the VMTH is the standard 1–9 range where a score of 5 is the goal [22]. Typically, the clinician assigns the BCS at the time of a patient's visit to the hospital. For this study the BCSs at the time of diagnosis (or clinical signs) of neutered dogs with joint disorders were compared with BCSs of neutered dogs without the disorder at an age that fell within the range representing 80 percent of the ages of dogs with the disorder at the time of diagnosis. The BCSs were compared between neutered dogs with and without joint disorders for the disorders that were significantly increased in incidence over that of intact dogs and for just the neuter periods where there were such differences. For the few joint disorders associated with neutering at one year or beyond, the BCSs were not included for comparison to maintain uniformity across comparisons. The data are represented as medians to reduce the impact of outliers.

### Statistical Analyses

While the study set out to estimate incidence rates of each disease related to age at neutering, patients were diagnosed at different ages and with differing durations of the disease as well as varying years at risk from the effects of gonadal hormone removal. Cox proportional hazard models (CPH) [23], [24] were used to test for group differences with respect to the hazard of a disease while adjusting for the time of neutering and the animal's age at diagnosis. All analyses were run using the SAS software package, version 9.3. Post hoc comparisons among the subgroups were based on least squares means of the hazard within each subgroup. In the Results section the *p*-values were based on these proportional hazard models. For all statistical tests the two-tailed statistical level of significance was set at *p*<0.05.

### Data Availability

In compliance with journal policy the final dataset used for statistical analyses, with the client information removed for confidentiality, is publically available at figshare.com: http://dx.doi.org/10.6084/m9.figshare.1038819.

## Results

With regard to joint disorders and cancers, the incidence rates at various neuter ages were much more pronounced in the Golden Retrievers than in the Labrador Retrievers. Therefore, results will be presented first for the Golden, and then the Labrador, with the two breeds contrasted. For joint disorders, BCSs are reported for those that differed significantly from the intact dogs, only for the neuter periods where the differences occurred. The mean age of diagnosis of joint disorders and cancers for each sex and breed is given to the nearest 0.5 years.

### Golden Retriever Males: Joint Disorders

Figure 1-A presents the incidence of dogs having at least one of the joint disorders. The incidence of at least one joint disorder occurring in intact males was 5 percent. At neuter age <6 mo., at least one of the joint disorders occurred in 27 percent of the males, or five times the incidence of intact males (*p*<0.0001). At neuter age 6–11 mo., this incidence was 14 percent or almost three times that of intact males (*p*<0.005). In the 2–8 year neutering period there was a moderate rise in this measure to double that of intact males (*p* = 0.02).

As shown in Figure 1-A and in Table 2, the main joint disorder related to neutering in males was HD, which was significantly higher than that of intact males for the <6 mo. and 6–11 mo. neuter periods (*p*<0.001; *p*<0.05, respectively). The mean age of diagnosis of HD in males was 4 years. The other important joint disorder was CCL, which was never diagnosed in intact males, and was significantly higher than intact males in the <6 mo. and 6–11 mo. neuter periods (*p*<0.001; *p* = 0.004, respectively). The mean age of diagnosis of this joint disorder in males was 5 years. In this breed the occurrence of ED was relatively minor compared with the other joint disorders and not significantly above that of intact males for any neuter period. When it did occur, mean age of diagnosis of ED was 2.5 years.

The median BCS of neutered males with HD was 6.0, and the median BCS of neutered males without HD was 5.5. In intact males with and without HD the median BCS was 5. For neutered males with CCL, the median BCS was 5.5 and for neutered males without CCL, 6.0. In intact males without CCL the median BCS was 5.0.

### Golden Retriever Males: Cancers

Figure 2-A presents the incidence in dogs having at least one of the cancers followed. The level in the intact males was 11 percent. At neuter ages <6 mo. and 6–11 mo. the occurrence of one or more cancers was 15–17 percent, but not significantly different than intact males. However, as Table 3 reveals, the main cancer elevated by neutering in males, LSA, reached 11.5 percent at the 6–11 mo. period, significantly higher than the 4 percent level of intact males (*p* = 0.007). The mean age of diagnosis of LSA in males was 5.5 years.

### Golden Retriever Females: Joint Disorders

Figure 1-A portrays the incidence of dogs having at least one of the joint disorders at different neuter periods. The incidence of at least one joint disorder occurring in intact females was 5 percent, virtually the same as males. At neuter age <6 mo. at least one of the joint disorders occurred in 20 percent of dogs, four times that of the intact females (*p*<0.001). At the 6–11 mo. neuter age, 13 percent had at least one joint disorder, which was over twice that of intact females, but did not reach significance.

As shown in Table 2, the main joint disorders related to neutering females at the <6 mo. period were HD and CCL, occurring at 10–11 percent. The occurrence of HD did not reach significance compared with intact females (4 percent), but CCL, which was not seen in any of the intact females, was significantly higher at the <6 mo., 6–11 mo. and 2–8 year neuter periods (*p*<0.001 to *p* = 0.03). The mean age of diagnosis of CCL in females was 5.5 years. As with males, the occurrence of ED in neutered females was not significant over that of intact females. The mean age of diagnosis of ED in females, when it did occur, was 1.5 years.

The median BCS of neutered females with CCL was 6.0 and the median BCS of the neutered females without CCL was 5.5. In intact females without CCL the median BCS was 5.0.

### Golden Retriever Females: Cancers

Figure 2-A presents the incidence of females having at least one of the cancers where the incidence of cancers in intact females was just 3 percent. The increase in cancers over all the neuter periods ranged from 8 to 14 percent. Combining all of the neuter periods beyond 6 mo. (to have a larger data set for analyses), the elevated incidence level across all these neuter periods was significantly higher than that of intact females (*p* = 0.049). The results reveal that neutering through 8 years of age increases the risk of acquiring at least one of the cancers to a level 3–4 times that of leaving the female dog intact.

Examination of Table 3 shows that the main cancer resulting from neutering females at <6 mo. and 6–11 mo. was LSA where at 6–11 mo. the increased risk over that of intact females reached significance (*p* = 0.014). The mean age of diagnosis of LSA in females was 5.5 years. The main cancer that was increased at the 2–8 year period of neutering was MCT (*p* = 0.013). The occurrence of HSA, although increased by neutering beyond 1 year, did not reach significance over intact females. The mean age of diagnosis of both MCT and HSA in females was 6.5 years.

The occurrence of MC was not seen in any of the intact females. This cancer was seen only in dogs neutered in the 2–8 year period where the incidence was 3.5 percent. The occurrence of pyometra in intact females was 1.8 percent, which was diagnosed at the mean age of 6 years.

### Labrador Retriever Males: Joint Disorders

Figure 1-B illustrates the incidence of males having at least one of the joint disorders. The only neuter period where this measure was significantly increased above the 5 percent level of intact males, was at <6 mo., where this measure was 12.5 percent (*p* = 0.014). Examining the joint disorders individually (Table 4), HD was not increased by neutering at any time. However, at the <6 mo. neuter period, both CCL and ED were significantly increased over that of intact males (*p* = 0.02; 0.02). For ED, there was a moderate increased risk with the 2–8 year neuter period to about 2 percent compared with the low 0.57 percent incidence in intact males (*p* = 0.006). The mean age of diagnosis of ED in males was 3 years, considerably less than that for CCL, which was 4.5 years.

The median BCS of neutered males with CCL was 6.0 and the median BCS of the neutered males without CCL was 5.0. In intact males with CCL the median BCS was 6.0 and for intact males without CCL the median BCS was 5. The median BCS of neutered males with ED was 6.5 and the median BCS of the neutered males without ED was 5.0. In intact males with and without ED the BCS was 5.0.

### Labrador Retriever Males: Cancers

The underlying rate of intact males having at least one of the cancers was 4.6 percent. Neutering at any age period had virtually no effect on this measure of cancer occurrence above the level of intact males (Figure 2-B and Table 5).

### Labrador Retriever Females: Joint Disorders

As portrayed in Figure 1-B, at neuter periods <6 mo. and 6–11 mo. the risk of dogs having at least one of the joint disorders increased to about double the 5 percent level of intact females (*p* = 0.044; 0.043). In contrast to male Labradors, the females seemed to be vulnerable to the effects of early neutering on HD but not on ED. The neutering effects on HD were evident through 1 year, where the incidence was 4–5 percent compared to 1.5 percent in intact females (Table 4) (*p* = 0.02–0.046). The mean age of diagnosis of HD was 3.5 years, and for ED, 2.5 years. As in male Labradors, CCL in females was increased by early neutering, but in this sex, not significantly so. The mean age of diagnosis of CCL in females was 5.5 years.

The median BCS of neutered females with HD was 5.5, and the median BCS of neutered females without HD was 5.5. In intact females with HD the median BCS was 7 and for those without HD the median BCS was 5.0.

### Labrador Retriever Females: Cancers

As seen in Figure 2-B, the underlying rate of intact females having at least one cancer of those tracked was 3.2 percent, close to that of males. In contrast to female Goldens, the only increase in the incidence of dogs having at least one cancer, was with the 2–8 year neuter period where the incidence was modestly increased to 5.6 percent (*p* = 0.03), a reflection of the increased occurrence of LSA and MCT (Table 5). The mean age of diagnosis of these two cancers in females was 5.5 and 6.5 years, respectively.

With regard to MC, only 1.4 percent of the intact females were diagnosed with MC. With the 2–8 year neuter period MC was diagnosed in 2 percent of females. Pyometra was diagnosed in just less than 4 percent of intact females. The mean age of diagnosis of pyometra was 5.5 years.

## Discussion

Both the Golden Retriever and Labrador Retriever are very popular breeds that have found wide acceptance as family pets and as service dogs for those with disabilities. The two breeds are similar in body size, conformation and in behavioral characteristics [25], and they share a similar developmental background as upland game retrievers. Using the same database and methodology, the two breeds were contrasted with regard to the effects of neutering on three joint disorders (HD, CCL, ED) and three cancers (LSA, HSA, MCT). In addition to reporting the occurrence of the three joint disorders and the three cancers, an analysis of cases with at least one of the joint disorders, or at least one of the cancers, was plotted graphically (Figures 1 and 2). The findings on the Golden Retriever closely resemble the picture presented in the earlier study drawn from this same database with a somewhat smaller data set [14].

The present study reveals that the breeds respond very differently to the effects of neutering on joint disorders and certain devastating cancers. With regard to the occurrence of one or more joint disorders, in Golden Retrievers, neutering at <6 mo. resulted in an incidence of 27 percent in males and 20 percent in females, 4–5 times the 5 percent level for intact males and females. In male and female Labrador Retrievers, with the same underlying occurrence of joint disorders in intact dogs, neutering at <6 mo. resulted in an incidence of 11–12 percent for one or more joint disorders, roughly double that of intact males and females. Thus, for both breeds, neutering at the standard <6 mo. period markedly and significantly increased the occurrence of joint disorders, although the increase was worse in the Golden than the Labrador. A difference in the specific joints affected was that in male Goldens HD and CCL were mostly increased, but in male Labradors CCL and ED were increased. The effects of neutering in the first year of a dog's life, especially in larger breeds, undoubtedly reflects the vulnerability of joints to delayed closure of long-bone growth plates from gonadal hormone removal [26], [27]. Differences in the two breeds studied here could be due to differences in sensitivities of the growth plates to gonadal hormone removal.

The BCSs in neutered dogs with the different joint disorders were compared with neutered dogs without the joint disorders. Although dogs with the disorders were expected to have a modestly higher BCS as a function of reduced activity from painful joints, the issue of concern was if those with a joint disorder had a consistently and markedly higher BCS than comparable neutered dogs without a joint disorder. The BCS comparisons revealed variable differences, in the range of 0.5 to 1.0 (except for ED in male Labradors where the difference was 1.5). The general picture of BCSs of neutered dogs with joint disorders being usually, but not always, a bit higher than the BCSs of neutered dogs without joint disorders, is consistent with the perspective that the increase in joint disorders in neutered dogs is primarily due to the effect of gonadal hormonal removal on bone growth plates and not to greater weight on the joints.

Data on the effects of neutering on the occurrence of cancers in the two breeds also reveal important breed differences. In both breeds the occurrence of one more cancers in intact dogs ranged from 3 to 5 percent, except for Golden Retriever males where the level in intact dogs was 11 percent. In Golden Retriever females neutering females at any neuter period beyond 6 months elevated the risk of one or more cancers to 3 to 4 times the level of intact females (Figure 2). In male Golden Retrievers neutering appeared to have little effect in the occurrence of one or more of the three cancers. An exception was LSA that was increased significantly at the <6 mo. period. In both male and female Labrador Retrievers, neutering at any period appeared to have little effect in increasing cancers.

The striking effect of neutering in female Golden Retrievers compared to male and female Labradors, and male Golden Retrievers, suggests that for this gender and breed the presence of gonadal hormones has a protective effect against cancers over most years of the dog's life. This may reflect a particular sensitivity of receptor sites of some potentially metastatic cancer cells to gonadal hormone removal and/or prolonged levels of the gonadotropin hormone, follicle stimulating hormone [28]. Gonadotropin receptors have been identified in some extragonadal tissues. For example, in the dog these receptor sites have been found in the skin [29] and urinary tract [30]. Treatment of one or more of these cancers by a receptor-site blocking agent may be worth exploring. The relatively high occurrence of one or more of these cancers in intact male Goldens, coupled with the relative absence of an effect of neutering, except with regard to LSA, points to a relatively high underlying rate of cancer occurrence in this gender and breed that is not affected by gonadal hormone removal.

The findings presented here are clinically relevant in two realms. For dog owners of the popular Golden Retrievers and Labrador Retrievers, the study points to the importance of acquiring information needed to decide if, and when, to neuter. Aside from avoiding increased risks of joint disorders and cancers, there is an indication that age-related cognitive decline could be accelerated by neutering [31]. This is particularly relevant for service dogs where active cognition is important for the expected tasks.

The findings of this study also have important implications for investigators looking for canine models for research on various forms of cancer [32], [33]. For some cancers of interest, not only may breeds vary in predisposition but also the possibility of interactions between gender, gonadal hormone influences, and timing of gonadal hormone alteration should be taken into account in selecting the model and in investigating causal factors to be explored.
